# Breast fibroadenomas are not associated with increased breast cancer risk in an African American contemporary cohort of women with benign breast disease

**DOI:** 10.1186/s13058-018-1027-6

**Published:** 2018-08-09

**Authors:** Asra N. Shaik, Julie J. Ruterbusch, Eman Abdulfatah, Resha Shrestha, M. H. D. Fayez Daaboul, Visakha Pardeshi, Daniel W. Visscher, Sudeshna Bandyopadhyay, Rouba Ali-Fehmi, Michele L. Cote

**Affiliations:** 1 Department of Oncology, Wayne State University School of Medicine, 4100 John R Street, MM04EP, Detroit, MI 48201 USA; 2 Department of Pathology, Wayne State University School of Medicine, Detroit, MI USA; 3 Department of Laboratory Medicine and Pathology, Mayo Clinic, Rochester, MN USA; 4 Barbara Ann Karmanos Cancer Institute, Detroit, MI USA

**Keywords:** Benign breast disease, Breast cancer, Risk, African American

## Abstract

**Background:**

Fibroadenomas are common benign breast lesions, and studies of European American women indicate a persistent, increased risk of breast cancer after diagnosing a fibroadenoma on biopsy. This association has not been independently assessed in African American women, despite reports that these women are more likely to present with fibroadenomas.

**Methods:**

The study cohort included 3853 African American women with a breast biopsy completed between 1997 and 2010 in metropolitan Detroit. Biopsies were microscopically reviewed for benign breast lesions, including fibroadenoma, proliferative disease, and atypia. Risk of breast cancer within the cohort was estimated using relative risk ratios and 95% CIs calculated using multivariable log-binomial regression. Relative risk of breast cancer in this cohort compared with African American women in the broader metropolitan Detroit population was estimated using standardized incidence ratios (SIRs).

**Results:**

Fibroadenomas occurred more frequently in biopsies of younger women, and other types of benign breast lesions were less likely to occur when a fibroadenoma was present (*p* = 0.008 for lobular hyperplasia; all other *p* values < 0.01). Unlike women with other benign lesions (SIR, 1.41; 95% CI, 1.20, 1.66), women with fibroadenomas did not have an increased risk of developing breast cancer compared with the general population (SIR, 0.94; 95% CI, 0.75, 1.18). Biopsies that indicated a fibroadenoma were associated with a reduced risk of breast cancer after adjusting for age at biopsy, proliferation, and atypia (relative risk, 0.67; 95% CI, 0.48, 0.93) compared with biopsies without a fibroadenoma.

**Conclusions:**

These findings have important implications for breast cancer risk models and clinical assessment, particularly among African American women, in whom fibroadenomas are common.

**Electronic supplementary material:**

The online version of this article (10.1186/s13058-018-1027-6) contains supplementary material, which is available to authorized users.

## Background

Over 1.5 million breast biopsies are pathologically assessed annually in the United States, indicated by abnormal mammography findings or patient complaints [1]. Most biopsies are not malignant, but instead exhibit a number of pathological lesions that constitute benign breast disease (BBD). Biopsies that exhibit proliferative disease or cellular atypia, as defined by Dupont and Page criteria, are consistently associated with increases in breast cancer risk [2–4]. These pathologic criteria have been included in risk assessment models to identify women at high risk of developing breast cancer. Several current risk assessment models, including the frequently used Breast Cancer Risk Assessment Tool, incorporate information on the number of prior biopsies and the presence of atypia on biopsy, but they do not account for other BBD lesions that may independently increase breast cancer risk [5]. Reliable estimates of breast cancer risk associated with individual lesions can improve risk models, allowing physicians to better identify women at high risk of developing breast cancer who may benefit from additional screening or chemoprevention.

**Fig. 1:**
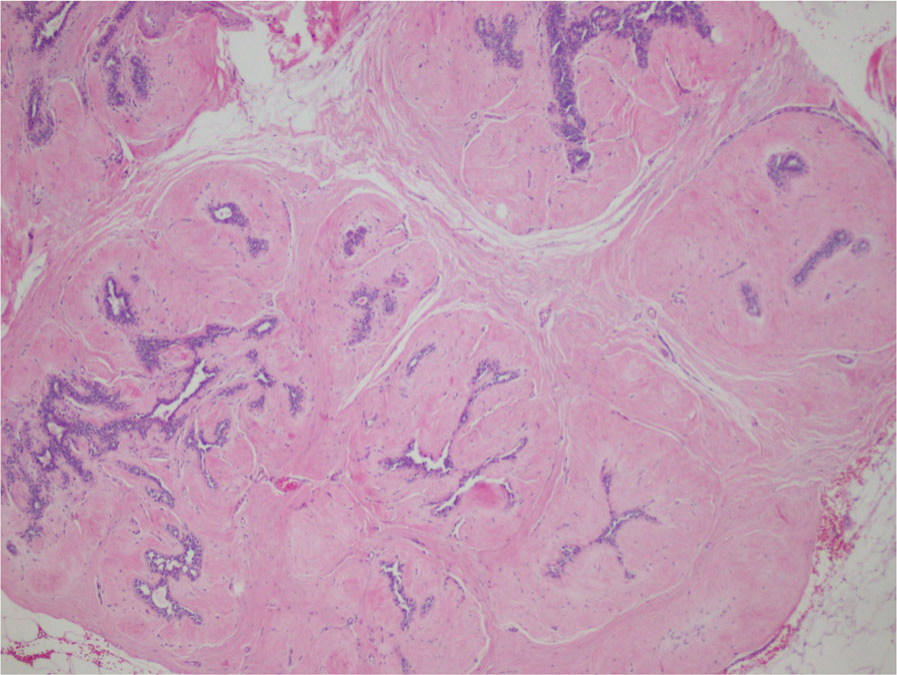
Fibroadenoma. Fibroadenomas are benign tumors of stromal and epithelial tissue that are typically well-circumscribed and mobile within the tissue. The fibroadenoma shown here exhibits purple epithelial tissue surrounded by pink fibrotic stromal tissue (H&E stain; original magnification 100 ×)

One type of BBD, fibroadenomas, are well-circumscribed benign tumors of epithelial and stromal tissue (Fig. 1) [6]. Breast fibroadenomas most frequently occur in women in their 20s [6] but can occur at any age; it is estimated that 10% of women have breast fibroadenomas [7]. A recent meta-analysis of 11 studies reported an increase in breast cancer risk by 41% (1.41; 95% CI, 1.11–1.80) for women diagnosed with a fibroadenoma compared with women without fibroadenoma on biopsy; however, significant statistical heterogeneity was also reported with this estimate [8]. Furthermore, the studies in this meta-analysis were primarily in European ancestral populations, and the majority were studies done prior to the widespread use of screening mammography in the 1980s. Although African American women experience a higher incidence and recurrence of fibroadenomas at a younger age [9, 10], breast cancer risk associated with this lesion has not been independently assessed in this population of women.

African American women have a 42% higher breast cancer mortality rate than European American women [11], a burden that stems partly from differences in tumor biology. African American women are more likely to develop breast cancer at a younger age [12–14] and more likely to be diagnosed with aggressive tumors characterized by high molecular grade [15, 16] and lack of hormone receptors [13, 15, 16]. Despite this survival disparity, prior investigations on BBD and breast cancer risk focused on mostly European American cohorts. The goal of this study is to examine in a contemporary cohort whether breast cancer risk associated with fibroadenoma differs for African American women, a population more likely to present with fibroadenomas and more likely to develop aggressive breast cancers that respond poorly to treatment.

## Methods

### Study population

African American women with their first benign breast biopsies conducted between 1997 and 2010 were identified using University Pathology Group (UPG; Detroit, MI, USA) records. UPG provides pathology services to several hospitals in metropolitan Detroit. Women aged 18 to 84 years at the time of benign breast biopsy were eligible for this institutional review board-approved study. Exclusionary criteria included a diagnosis of invasive or in situ breast carcinoma before or within 6 months of the breast biopsy, a history of mastectomy or reduction mammoplasty, lipoma, fat necrosis, epidermal cysts, hematoma, accessory structure, phyllodes tumor, or a lymph node biopsy without breast tissue. For this type of study, the Wayne State University Institutional Review Board determined that formal consent was not required.

### Histological review

Core needle and excisional benign biopsies were microscopically reviewed by blinded study pathologists (RAF, SB) using original H&E-stained slides. Slides from the first biopsy were assessed for the presence of 12 pathologic lesions, including apocrine metaplasia, calcifications, columnar alterations, cysts, duct ectasia, ductal hyperplasia, fibroadenoma, fibrosis, intraductal papilloma, lobular hyperplasia, radial scars, and sclerosing adenosis. The biopsies were additionally categorized into three groups using criteria described by Dupont and Page [2] based on the presence of proliferative disease and cellular atypia. Biopsies classified as showing atypia and a random sample of all other biopsies were reassessed by a blinded study pathologist at the Mayo Clinic (DWV). Breast biopsies that could not be assessed for fibroadenoma presence were excluded from analysis (*n* = 23).

### Cancer ascertainment

Women who developed breast cancer were identified through hospital medical records and also through the use of the Metropolitan Detroit Cancer Surveillance System (MDCSS), a founding member of the National Cancer Institute’s Surveillance, Epidemiology, and End Results (SEER) program. MDCSS collects cancer incidence, treatment, and survival data in the tricounty metropolitan Detroit area. Use of both data sources allowed the identification of cancers in women residing in the entire tricounty metropolitan Detroit area. Women were matched between UPG records and MDCSS using name, date of birth, and/or Social Security number; follow-up information was complete to December 31, 2015. Median length of follow-up was 13.3 years (range, 0.5–19.0 years); median time to breast cancer diagnosis was 6.6 years (range, 0.7–18.5 years).

### Statistical analysis

Associations between fibroadenoma and other benign lesions were examined using chi-squared tests. Relative risks of breast cancer associated with biopsies, with or without fibroadenoma, were estimated using age-adjusted standardized incidence ratios (SIRs) calculated from SEER estimates of cancer incidence in African American women in MDCSS from 1999 to 2014. Risk of breast cancer associated with fibroadenomas relative to other nonfibroadenoma BBD was examined within the cohort by relative risk ratios and 95% CIs calculated using multivariable log-binomial regression and adjusting for age at biopsy. Regression models were further adjusted using backwards selection based on Bayesian information criteria. Models were stratified by age (below or above 50 years) to estimate risk based on likely menopausal status. Time to breast cancer diagnosis was assessed using competing risk analysis with death considered as a competing risk.

## Results

### Distribution of BBD features and characteristics by fibroadenoma status

A total of 3845 benign breast biopsies were assessed in this African American cohort, 1798 (47%) of which were diagnosed with fibroadenoma. Fibroadenomas showed high concordance between pathologists (86.9%; Cohen’s κ = 0.7022). Women with a fibroadenoma on biopsy were more likely to be under the age of 40 years at biopsy (31.9%) than women without a fibroadenoma on biopsy (18.9%) (*p* < 0.001) (Table 1). The presence of a fibroadenoma was associated with the absence of all other benign breast lesions assessed on biopsy (*p* = 0.008 for lobular hyperplasia; all other *p* < 0.001) (Table 1 and Additional file 1: Tables S1 and S2). Additionally, biopsies with a fibroadenoma were less likely to be classified as proliferative disease (25.0%) or proliferative disease with atypia (1.3%) than biopsies without a fibroadenoma (51.5% and 6.1%, respectively).

**Table 1:** Distribution of benign breast features and other characteristics by fibroadenoma status

Characteristic	Status, *n* (%)^a^		*P* value^b^
	No fibroadenoma 2047 (53.2)	Fibroadenoma 1798 (46.8)	
Age at benign biopsy, years			< 0.001
< 40	387 (18.9)	573 (31.9)	
40–49	692 (33.8)	582 (32.4)	
50–59	577 (28.2)	374 (20.8)	
60–69	249 (12.2)	164 (9.1)	
70+	142 (6.9)	105 (5.8)	
Biopsy type			< 0.001
Excisional	826 (40.4)	536 (30.8)	
Core needle	1221 (59.6)	1262 (70.2)	
Apocrine metaplasia			< 0.001
Absent	1202 (58.7)	1401 (82.3)	
Present	845 (41.3)	301 (17.7)	
Ductal hyperplasia			< 0.001
Absent	1272 (62.1)	1365 (80.6)	
Present	775 (37.9)	329 (19.4)	
Lobular hyperplasia			0.008
Absent	2012 (98.3)	1662 (99.3)	
Present	34 (1.7)	11 (0.7)	
Calcifications			< 0.001
Absent	1209 (59.1)	1229 (70.8)	
Present	837 (40.9)	507 (29.2)	
Cysts			< 0.001
Absent	970 (47.4)	1339 (78.9)	
Present	1076 (52.6)	359 (21.1)	
Duct ectasia			< 0.001
Absent	1652 (80.7)	1546 (91.0)	
Present	394 (19.3)	152 (9.0)	
Fibrosis			< 0.001
Absent	648 (31.7)	1031 (63.8)	
Present	1397 (68.3)	586 (36.2)	
Intraductal papilloma			< 0.001
Absent	1662 (81.2)	1629 (96.1)	
Present	385 (18.8)	66 (3.9)	
Sclerosing adenosis			< 0.001
Absent	1416 (69.2)	1404 (82.7)	
Present	630 (30.8)	294 (17.3)	
Columnar alterations			< 0.001
Absent	1302 (63.6)	1439 (84.7)	
Present	744 (30.8)	259 (15.3)	
Radial scar			< 0.001
Absent	1975 (96.5)	1665 (98.6)	
Present	71 (3.5)	23 (1.4)	
Dupont and Page criteria			< 0.001
Nonproliferative disease	868 (42.4)	1325 (73.7)	
Proliferative disease without atypia	1054 (51.5)	450 (25.0)	
Proliferative disease with atypia	125 (6.1)	23 (1.3)	
Developed breast cancer			< 0.001
No	1902 (92.9)	1722 (95.8)	
Yes	145 (7.1)	76 (4.2)	

^a^Numbers may not sum to the total number of patients if features could not be assessed on biopsy

^b^χ^2^ test comparing distribution of features across absence or presence of fibroadenoma on biopsy

### Breast cancer risk compared with population level risk

Overall, this cohort of women exhibited an increased incidence of approximately 20% (SIR, 1.19; 95% CI, 1.05–1.36) of breast cancer compared with the general African American population in metropolitan Detroit (Table 2). Stratifying the cohort by presence of fibroadenoma on biopsy revealed that breast cancer incidence associated with fibroadenoma was indistinguishable from population level (SIR, 0.93; 95% CI, 0.75–1.17), but the breast cancer incidence associated with the absence of fibroadenoma on biopsy was significantly higher than population level (SIR, 1.40; 95% CI, 1.19–1.65).

**Table 2:** Risk of breast cancer compared with population level risk

	Standardized incidence ratio^a^	95% confidence interval
Population rate	Ref	
Entire BBD cohort (*N* = 221 cancers)	1.19	1.05–1.36
Biopsy without fibroadenoma (*N* = 145 cancers)	1.40	1.19–1.65
Fibroadenoma (*N* = 76 cancers)	0.93	0.75–1.17

^a^Standardized incidence ratio compares the observed number of breast cancers that developed in the study to the number expected on the basis of the Detroit surveillance, epidemiology, and end results data for African American women of a similar age and calendar period

### Breast cancer risk within the BBD cohort

Adjusting for age at biopsy alone, the presence of fibroadenoma was associated with a reduced breast cancer risk (relative risk [RR], 0.64; 95% CI, 0.45–0.85) compared with the absence of fibroadenoma within the BBD cohort (Table 3). When the model was fully adjusted for age at biopsy, proliferation, and atypia, fibroadenoma was still associated with a reduced risk (RR, 0.67; 95% CI, 0.48–0.93) of developing breast cancer. Fibroadenoma diagnosed in women under the age of 50 years was associated with a decrease in breast cancer risk after adjusting for age at biopsy, proliferation, and cellular atypia (RR, 0.58; 95% CI, 0.34–0.96). Fibroadenoma diagnosed in women aged 50 years or older also showed a reduction in breast cancer risk but failed to reach statistical significance after adjusting for age at biopsy, proliferation, and cellular atypia (RR, 0.79; 95% CI, 0.52–1.19).

**Table 3:** Relative risk of breast cancer by fibroadenoma status

	Age-adjusted relative risk^a^ (95% CI)	*P* value^b^	Fully adjusted relative risk^c^ (95% CI)	*P* value^b^
No fibroadenoma on biopsy	Ref		Ref	
Fibroadenoma	0.64 (0.48, 0.85)^d^	0.003	0.67 (0.48, 0.93)^e^	0.017
Under age 50 years				
No fibroadenoma on biopsy	Reference		Reference	
Fibroadenoma	0.71 (0.45, 1.11)^f^	0.133	0.58 (0.34, 0.96)^g^	0.037
Age 50 years or older				
No fibroadenoma on biopsy	Reference		Reference	
Fibroadenoma	0.68 (0.46, 0.98)^h^	0.042	0.79 (0.52, 1.19)^i^	0.275

^a^Multivariable logistic regression model adjusting for age at biopsy

^b^Wald test statistic

^c^Multivariable logistic regression model adjusting for age, proliferative disease, and cellular atypia at biopsy

Number at risk: ^d^3845, ^e^3761, ^f^2234, ^g^2071, ^h^1611, ^i^1536

### Cumulative incidence of cancers in subgroups

**Fig. 2:**
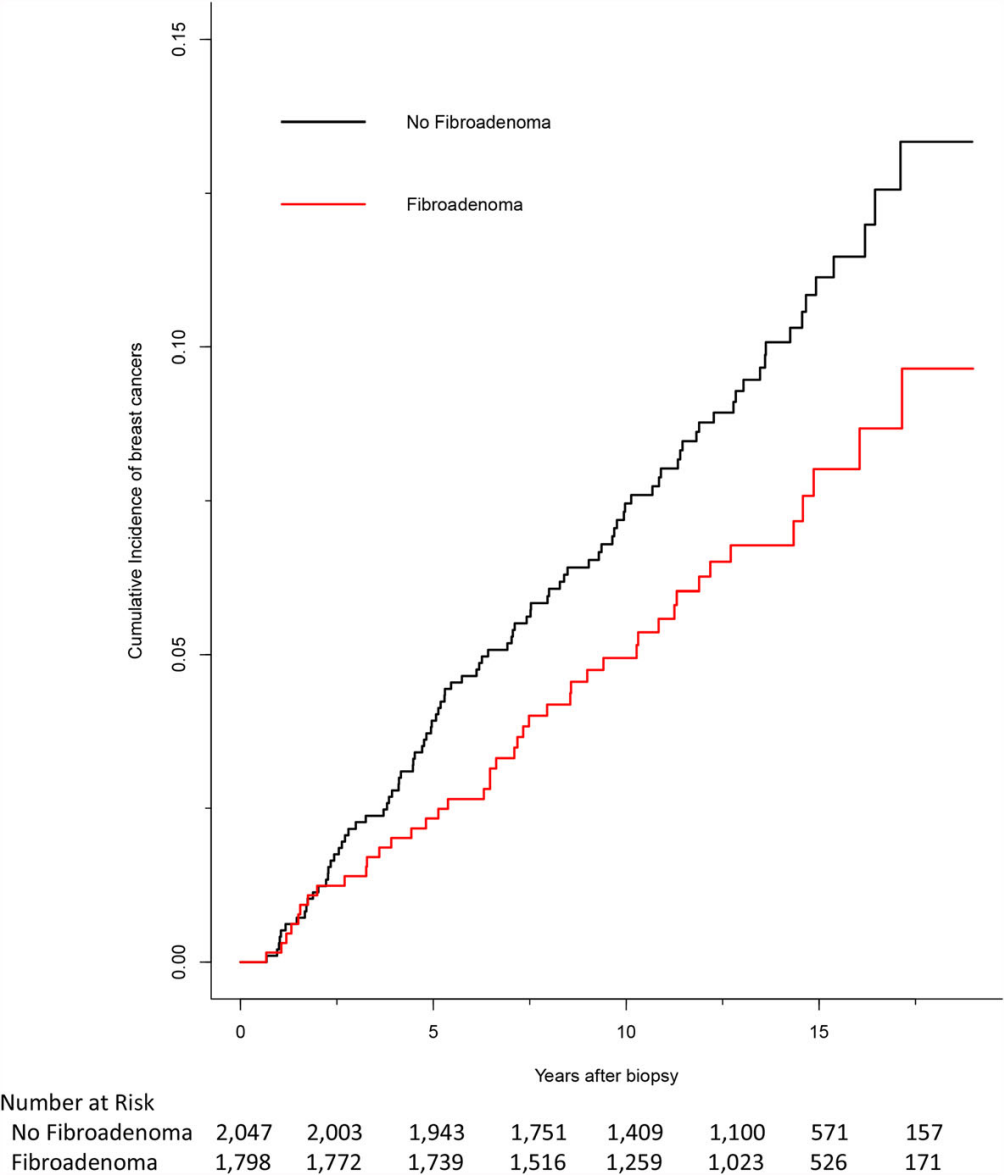
Cumulative incidence of breast cancers over study period. Women with biopsies that indicated fibroadenomas accumulated fewer breast cancers over the study period than women whose biopsies did not indicate fibroadenomas. *p* < 0.001 by Fine and Gray test

Women with fibroadenoma on biopsy accumulated fewer breast cancers over the study period than women without fibroadenoma on biopsy (Fig. 2) (*p* < 0.001 by Fine and Gray test). Stratifying by likely menopausal status by age indicated the incidence of breast cancers was lower in women under the age of 50 years than in women aged 50 years or older (data not shown). In both strata, women with fibroadenoma on biopsy accumulated fewer cancers over the study period than women without fibroadenoma on biopsy (Fine and Gray test, *p* = 0.014 for under age 50 years and *p* = 0.059 for ages 50 years and older).

## Discussion

We report findings in a contemporary cohort of African American women who have had a breast biopsy that show those with a fibroadenoma observed on biopsy are not at increased risk of subsequent breast cancer compared with the general population of African American women. When compared with all benign biopsies, biopsies that indicated a fibroadenoma were associated with a reduced risk of breast cancer that remains significant even after adjusting for age, proliferative disease, and atypia. These findings suggest that current breast cancer risk models that incorporate benign biopsies without considering the pathological lesion overestimate risk in African American women who have fibroadenomas on biopsy. Given that fibroadenomas were identified in nearly half of all breast biopsies in this population and were the only lesion identified in 19% of all biopsies, these findings represent a significant clinical population.

Our investigation suggests that biopsies indicating fibroadenoma exhibit a reduced risk of breast cancer compared with all other BBD biopsies, contrary to most other studies’ estimates of increased risk of breast cancer [8]. Discordant risk estimates between our investigation and those from other studies may reflect differences in race, age, and period of cohorts used. The Nashville group [17], which found a significant increase in breast cancer risk with fibroadenoma (SIR, 1.61; 95% CI, 1.30–2.00) compared with the Connecticut Tumor Registry, studied European American women diagnosed with a fibroadenoma between 1950 and 1968. The Mayo Clinic BBD cohort [18] studied European American women diagnosed with fibroadenoma between 1967 and 1991 and found modest increases breast cancer risk with fibroadenoma (SIR, 1.60; 95% CI, 1.38–1.85) compared with biopsies without fibroadenoma (SIR, 1.50; 95% CI, 1.39–1.62). A BBD cohort from Henry Ford Health System (HFHS) [19], where women with fibroadenomas on biopsy had a decreased odds (OR, 0.55; 95% CI, 0.39–0.77) of developing breast cancer compared with women without fibroadenoma on biopsy, more closely approximating our risk estimates, studied a mixed cohort of European American and African American women in metropolitan Detroit diagnosed between 1981 and 1994. However, it is unlikely that the differences in risk estimates are due solely to race: the HFHS group tested an interaction factor between race and BBD and did not find statistical significance [19].

Period effects may also contribute to variation in risk estimates. Inclusion criteria for BBD studies span from 1950 to 2010; thus, differences in risk estimates may also reflect the endogenous and exogenous exposures that varied over this period. Exogenous hormone use, including hormone replacement therapy and contraceptive use, have changed in frequency, dose, and formulation. Changes in exogenous hormone use can alter total estrogen exposure, a strong breast cancer risk factor, and influence risk estimates of tissue-based markers. Environmental exposures that vary over time and/or geographic areas can further add to risk estimate variation. Changes in the indication for biopsy are perhaps the most pertinent shift over these study periods: physicians are more likely to biopsy now than in the 1950s. Population uptake of mammography began in the 1970s [20], and screening technology has continued to improve since then [21, 22], leading to an increase in breast biopsy incidence. The adoption of core needle biopsies, which are less invasive than excisional biopsies, further increased the likelihood of a breast biopsy, especially in what are considered high-risk populations.

The strengths of our study stem from the cohort study design, where all breast biopsies were reexamined for benign lesions in a centralized and standardized manner by Wayne State University pathologists, and identification of breast cancers occurred through institutional medical records and then standardized for the region through use of the population-based SEER registry. This allowed for the identification of breast cancers among women who sought care outside of the hospitals served by the UPG. It should be noted there are limitations to our study. First, the population estimates used in the SIR analysis includes women who have been diagnosed with BBD in the metropolitan Detroit area; thus, the SIR may slightly underestimate the risk associated with breast cancer. Next, our assessment was limited to the presence or absence of fibroadenomas on breast biopsy, but there may be added value in assessing whether these fibroadenomas exhibit other BBD lesions. There are conflicting reports on the breast cancer risk associated with complex fibroadenomas (fibroadenomas that exhibit cysts, calcifications, sclerosing adenosis, and/or apocrine metaplasia) [17, 18]. Because of the high prevalence of fibroadenomas in this population, breast cancer risk associated with complex fibroadenoma should also be independently reviewed in African American women.

## Conclusions

Currently, a diagnosis of fibroadenoma requires no further intervention and is followed by a primary care physician or gynecologist unless the patient elects to have a mass removed, usually due to size of the tumor, recurrence, or pain [23, 24]. Because previous investigations of fibroadenoma on biopsy estimated an elevated risk of breast cancer that persists for 20 years [17], physicians may currently screen women with fibroadenomas frequently. Our study suggests that fibroadenomas do not increase risk of subsequent breast cancers. Ultimately, examining specific features of BBD will improve risk estimates used in breast cancer risk models, reduce patient anxiety, and improve management of fibroadenoma in the clinic by reducing overscreening and overtreatment of this population, both associated with potential patient harms and excessive resource allocation.

## Additional file

Additional file 1: **Table S1.** Distribution of benign breast features and other characteristics by fibroadenoma status in women under the age of 50 years. **Table S2.** Distribution of benign breast features and other characteristics by fibroadenoma status for women aged 50 years or older. (DOCX 32 kb)

## Abbreviations

BBD: Benign breast disease
FA: Fibroadenoma
HFHS: Henry Ford Health System
MDCSS: Metropolitan Detroit Cancer Surveillance System
RR: Relative risk
SEER: Surveillance, Epidemiology, and End Results program
SIR: Standardized incidence ratio
UPG: University Pathology Group
