# Supplementary material for: Breast fibroadenomas are not associated with increased breast cancer risk in an African American contemporary cohort of women with benign breast disease
Source: Breast Cancer Res. 2018 Aug 9;20:91. doi: 10.1186/s13058-018-1027-6 (PMC6085691; doi:10.1186/s13058-018-1027-6)
Supplement: Supplementary file 1 — Table S1. Distribution of benign breast features and other characteristics by fibroadenoma status in women under the age of 50 years. Table S2. Distribution of benign breast features and other characteristics by fibroadenoma status for women aged 50 years or older. (DOCX 32 kb) [file 13058_2018_1027_MOESM1_ESM.docx]

**Supplementary tables**

| **Table S1.** Distribution of benign breast features and other characteristics by fibroadenoma status for women under the age 50 | | | | |
| --- | --- | --- | --- | --- |
| Characteristic | | Status, N (%)^a^ |  | *P* value^b^ |
|  | | No Fibroadenoma  1079 (48.3) | Fibroadenoma  1155 (51.7) |  |
| Age at benign biopsy | |  |  | <0.001 |
|  | <40 | 387 (35.9) | 573 (49.6) |  |
|  | 40-49 | 692 (64.1) | 582 (50.4) |  |
| Apocrine Metaplasia | |  |  | <0.001 |
|  | Absent | 645 (59.8) | 905 (83.7) |  |
|  | Present | 434 (40.2) | 176 (16.3) |  |
| Ductal Hyperplasia | |  |  | <0.001 |
|  | Absent | 690 (63.9) | 876 (81.5) |  |
|  | Present | 389 (36.1) | 199 (18.5) |  |
| Lobular Hyperplasia | |  |  | 0.072 |
|  | Absent | 1069 (99.2) | 1065 (99.8) |  |
|  | Present | 9 (0.8) | 2 (0.2) |  |
| Calcifications | |  |  | <0.001 |
|  | Absent | 743 (68.9) | 960 (87.1) |  |
|  | Present | 336 (31.1) | 142 (12.9) |  |
| Cysts | |  |  | <0.001 |
|  | Absent | 526 (48.7) | 860 (79.9) |  |
|  | Present | 553 (51.3) | 217 (20.1) |  |
| Duct Ectasia | |  |  | <0.001 |
|  | Absent | 853 (79.1) | 977 (90.7) |  |
|  | Present | 226 (20.9) | 100 (9.3) |  |
| Fibrosis | |  |  | <0.001 |
|  | Absent | 323 (30.0) | 663 (64.9) |  |
|  | Present | 755 (70.0) | 359 (35.1) |  |
| Intraductal Papilloma | |  |  | <0.001 |
|  | Absent | 896 (83.0) | 1045 (97.1) |  |
|  | Present | 183 (17.0) | 31 (2.9) |  |
| Sclerosing Adenosis | |  |  | <0.001 |
|  | Absent | 712 (66.0) | 887 (82.4) |  |
|  | Present | 367 (34.0) | 190 (17.6) |  |
| Columnar Alterations | |  |  | <0.001 |
|  | Absent | 705 (65.3) | 931 (86.4) |  |
|  | Present | 374 (34.7) | 146 (13.6) |  |
| Radial Scar | |  |  | 0.003 |
|  | Absent | 1042 (96.6) | 1061 (98.6) |  |
|  | Present | 37 (3.4) | 15 (1.4) |  |
| Dupont and Page criteria | |  |  | <0.001 |
|  | Nonproliferative disease | 479 (44.4) | 861 (74.5) |  |
|  | Proliferative disease without atypia | 549 (50.9) | 290 (25.1) |  |
|  | Proliferative disease with atypia | 51 (4.7) | 4 (0.3) |  |
| Developed breast cancer | |  |  | 0.021 |
|  | No | 1027 (95.2) | 1122 (97.1) |  |
|  | Yes | 52 (4.8) | 33 (2.9) |  |

^a^Numbers may not sum to the total number of patients if features could not be assessed on biopsy
^b^χ^2^ test comparing distribution of features across absence or presence of fibroadenoma on biopsy

| **Table S2.** Distribution of benign breast features and other characteristics by fibroadenoma status for women aged 50 or older | | | | |
| --- | --- | --- | --- | --- |
| Characteristic | | Status, N (%)^a^ |  | *P* value^b^ |
|  | | No Fibroadenoma  968 (60.1) | Fibroadenoma  643 (39.9) |  |
| Age at benign biopsy | |  |  | 0.658 |
|  | 50-59 | 577 (59.6) | 374 (58.2) |  |
|  | 60-69 | 249 (25.7) | 164 (25.5) |  |
|  | 70+ | 142 (14.7) | 105 (16.3) |  |
| Apocrine Metaplasia | |  |  | <0.001 |
|  | Absent | 557 (57.5) | 496 (79.9) |  |
|  | Present | 411 (42.5) | 125 (20.1) |  |
| Ductal Hyperplasia | |  |  | <0.001 |
|  | Absent | 582 (60.1) | 489 (79.0) |  |
|  | Present | 386 (39.9) | 130 (21.0) |  |
| Lobular Hyperplasia | |  |  | 0.201 |
|  | Absent | 943 (97.4) | 597 (98.5) |  |
|  | Present | 25 (2.6) | 9 (1.5) |  |
| Calcifications | |  |  | 0.027 |
|  | Absent | 466 (48.2) | 269 (42.4) |  |
|  | Present | 501 (51.8) | 365 (57.6) |  |
| Cysts | |  |  | <0.001 |
|  | Absent | 444 (45.9) | 479 (77.1) |  |
|  | Present | 523 (54.1) | 142 (22.9) |  |
| Duct Ectasia | |  |  | <0.001 |
|  | Absent | 799 (82.6) | 569 (91.6) |  |
|  | Present | 168 (17.4) | 52 (8.4) |  |
| Fibrosis | |  |  | <0.001 |
|  | Absent | 325 (33.6) | 368 (61.8) |  |
|  | Present | 642 (66.4) | 227 (38.2) |  |
| Intraductal Papilloma | |  |  | <0.001 |
|  | Absent | 766 (79.1) | 584 (94.3) |  |
|  | Present | 202 (20.9) | 35 (5.7) |  |
| Sclerosing Adenosis | |  |  | <0.001 |
|  | Absent | 704 (72.8) | 517 (83.3) |  |
|  | Present | 263 (27.2) | 104 (16.7) |  |
| Columnar Alterations | |  |  | <0.001 |
|  | Absent | 597 (61.7) | 508 (81.8) |  |
|  | Present | 370 (38.3) | 113 (18.2) |  |
| Radial Scar | |  |  | 0.013 |
|  | Absent | 933 (96.5) | 604 (98.7) |  |
|  | Present | 34 (3.5) | 8 (1.3) |  |
| Dupont and Page criteria | |  |  | <0.001 |
|  | Nonproliferative disease | 389 (40.2) | 464 (72.2) |  |
|  | Proliferative disease without atypia | 505 (52.2) | 160 (24.9) |  |
|  | Proliferative disease with atypia | 74 (7.6) | 19 (3.0) |  |
| Developed breast cancer | |  |  | 0.049 |
|  | No | 875 (90.4) | 600 (93.3) |  |
|  | Yes | 93 (9.6) | 43 (6.7) |  |

^a^Numbers may not sum to the total number of patients if features could not be assessed on biopsy
^b^χ^2^ test comparing distribution of features across absence or presence of fibroadenoma on biopsy
